# Is neutrophil elastase the missing link between emphysema and fibrosis? Evidence from two mouse models

**DOI:** 10.1186/1465-9921-6-83

**Published:** 2005-07-26

**Authors:** Monica Lucattelli, Barbara Bartalesi, Eleonora Cavarra, Silvia Fineschi, Benedetta Lunghi, Piero A Martorana, Giuseppe Lungarella

**Affiliations:** 1 Department of Physiopathology & Experimental Medicine, University of Siena, 53100 Siena, Italy

## Abstract

**Background:**

The separation of emphysema from fibrosis is not as clear-cut as it was thought in early studies. These two pathologies may be present at the same time in human lungs and in mice either instilled with elastolytic enzymes or bleomycin or exposed to cigarette-smoke. According to a current view, emphysema originates from a protease/antiprotease imbalance, and a role for antiproteases has also been suggested in the modulation of the fibrotic process. In this study we investigate in experimental animal models of emphysema and fibrosis whether neutrophil elastase may constitute a pathogenic link between these two pathologies.

**Methods:**

This study was done in two animal models in which emphysema and fibrosis were induced either by bleomycin (BLM) or by chronic exposure to cigarette-smoke. In order to assess the protease-dependence of the BLM-induced lesion, a group mice was treated with 4-(2-aminoethyl)-benzenesulfonyl fluoride hydrochloride, a serine proteinase inhibitor active toward neutrophil elastase. Lungs from each experimental group were used for the immunohistochemical assessment of transforming growth factor-β (TGF-β) and transforming growth factor-α (TGF-α) and for determination of the mean linear intercept as well as the percent volume densities of fibrosis and of emphysematous changes. Additionally, the lungs were also assessed for desmosine content and for the determination of elastase levels in the pulmonary interstitium by means of immunoelectron microscopy.

**Results:**

We demonstrate that in BLM-treated mice (*i*) the development of elastolytic emphysema precedes that of fibrosis; (*ii*) significant amount of elastase in alveolar interstitium is associated with an increased expression of TGF-β and TGF-α; and finally, (*iii*) emphysematous and fibrotic lesions can be significantly attenuated by using a protease inhibitor active against neutrophil elastase.

Also, in a strain of mice that develop both emphysema and fibrosis after chronic cigarette-smoke exposure, the presence of elastase in alveolar structures is associated with a positive immunohistochemical reaction for reaction for both TGF-β and TGF-α.

**Conclusion:**

The results of the present study strongly suggest that neutrophil elastase may represent a common pathogenic link between emphysema and fibrosis. Proteases and in particular neutrophil elastase could act as regulatory factors in the generation of soluble cytokines with mitogenic activity for mesenchymal cells resulting either in emphysema or in fibrosis or both.

## Background

Lung emphysema and fibrosis are generally considered to be two diseases that totally differ in their morphological aspects and pathogenic mechanisms.

However, both these pathologies may be present at the same time in lungs of mice after cigarette-smoke exposure [1], in animals instilled intratracheally with BLM or other substances [2-5], as well as in human lungs [6]. In particular, in smokers and ex-smokers, centrilobular emphysema may be associated with some subsets of idiopathic interstitial pneumonias (IIP), namely "desquamative interstitial pneumonia" (DIP), "respiratory bronchiolitis-associated lung disease" (RB-ILD), and finally "idiopathic pulmonary fibrosis" (IPF) also characterized by the histological pattern of "usual interstitial pneumonia" (UIP). This has been clearly outlined in a recent document of the ATS/ERS defining the clinical manifestations, pathology and radiologic features of patients with IIP [7]. These data all together suggest a common pathway in the development of these two pathologies.

According to a current view, pulmonary emphysema originates from an imbalance between elastinolytic proteases and their naturally occurring inhibitors. In particular, neutrophil elastase (NE), and other elastolytic proteases, such as cathepsin G, and macrophage elastase are thought to be the main causative factors of tissue damage in this condition. This hypothesis is based on a mixture of evidence from animal models, broncho-alveolar lavage fluid (BALF) data, *in vitro *experiments, and from the high incidence of emphysema in homozygous subjects with a deficiency of α_l_-proteinase inhibitor (α_1_-PI) [8,9].

Recently, it has been reported that α_1_-PI, the secretory leukocyte protease inhibitor (SLPI), as well as the synthetic inhibitor of leukocyte elastase ONO-5046, significantly attenuate the fibrotic response to BLM in rodents [10-12]. In man, the inactivation of proteolytic enzymes may also be a critical event in normal repair, and as demonstrated in infants with respiratory distress syndrome, lack of antiprotease activity is associated with chronicity and development of fibrosis [13].

Thus, these studies suggest a significant role for the antiprotease screen not only in the development of pulmonary emphysema but also in the modulation of fibrotic lesions. This is further supported by studies carried out in BLM-challenged mice either with a genetic deficiency in α_1_-PI [4], or with a targeted deletion of the NE gene [14].

As previously reported by us, BLM administration induces alveolitis and fibrosis in α_1_-PI deficient mice. It also results in enlargement of air spaces that may be due either to loss of alveolar septa and/or retraction forces caused by the fibrotic process [4].

In this study we investigate whether NE may constitute a pathogenic link between emphysema and fibrosis. This was done in two animal models in which these two pathologies were induced either by BML or chronic exposure to cigarette smoke. In order to assess the protease-dependence of the BLM-induced lung lesion under our experimental conditions, a group mice was treated with 4-(2-aminoethyl)-benzenesulfonyl fluoride hydrochloride, a serine proteinase inhibitor fully active against NE [15].

## Methods

### Animals and Animal Studies

*Balb/c, C57 Bl/6 and DBA/2 *mice were supplied by Charles River (Calco, Italy). *C57 Bl/6J pa/pa (pallid) *mice were from our colony and were originally obtained from Jackson Laboratory (Bar Harbor, ME, USA). All animal experiments were conducted in conformance with the "Guiding Principles for Research Involving Animals and Human Beings" and approved by the Local Ethical Committee of the University of Siena.

#### Bleomycin Study

The study was carried out by using strains of mice with different levels of serum antielastase screen *(Balb/c, C57 Bl/6 and pallid*, with normal, intermediate and low screen, respectively) [16]. Male mice, weighing 20 to 25 g (8–10 wk old) were treated under ether anaesthesia with a single intratracheal instillation of 0.1 μg BLM (Rhone-Poulenc Rorer, Milano, Italy) in saline solution (50 μl) or with the same amount of saline. At 3, 7, 14, 21, 28 and 35 days after the treatment, animals were killed by pentobarbital sodium overdose and exsanguinated by cutting the abdominal aorta. Lungs were used for immunohistochemistry of TGF-β and TGF-α and for determination of the mean linear intercept (Lm) [17] as well as the percent volume densities of fibrosis Vv(f) and emphysematous changes Vv(e). Details of the morphometric assessment are given below. Lungs were also assessed for desmosine [18] and for determination of the elastase burden by immunoelectron microscopy [19].

#### Animal treatment with a synthetic serine-proteinase inhibitor

In order to assess the protease-dependence of the BLM-induced lung lesion under our experimental conditions two groups of ten *C57 Bl/6 *mice were treated with either 4-(2-aminoethyl)-benzenesulfonyl fluoride hydrochloride (Merck), a serine proteinase inhibitor, or with the vehicle (controls). This inhibitor (2.4 μg/μl saline) was continuously delivered at a rate of 0.5 μl/hr for two weeks by means of osmotic pumps (Alzet 2002, Alza Corporation, Palo Alto, CA). The pumps were implanted subcutaneously according the manufacturer's instructions, 24 hr before BLM-treatment. At 7 and 14 days after instillation with BLM, the lungs were excised, processed for histology, and used for morphometrical assessment of emphysematous and fibrotic lesions as well as for TGF-β and TGF-α immunohistochemistry.

#### Cigarette Smoke Study

Two months old male mice of the strain *DBA/2 *were exposed to either the smoke of 3 cigarettes/day (commercial Virginia filter cigarettes: 12 mg of tar and 0.9 mg of nicotine), 5 days/week, or to room air (controls), for various periods of time (from 1 to 6 months) as previously described in detail [20]. Groups of 8 animals were sacrificed during the chronic exposure period at various time intervals. The lungs were excised and processed for histology. Histological sections were stained with hematoxylin-eosin and Masson's trichrome. Morphometric assessment of emphysema included the determination of the Lm [17] and of the internal surface area (ISA) estimated by the Lm method at postfixation lung volume [21]. Fibrosis was assessed by point counting as Vv(f) as described below. Tissue sections were also stained for TGF-β, TGF-α and NE.

### Methodologies

#### Lung desmosine assay

To quantitate lung elastin, the lungs of each group were weighed, homogenised (1:5, w:v) and hydrolysed in 6 N HC1 before biochemical determinations. Desmosine was analysed on hydrolysates by means of an enzyme-linked immunosorbent assay essentially according to Cocci et al. [18]. Briefly, rabbit antiserum to desmosine-hemocyanin conjugate (Abl) (Elastin Product Company, Inc, Owensville, MO) was incubated with desmosine standard (0–30 ng) (Elastin Product Company, Inc, Owensville, MO) or with adequately diluted hydrolysates for 16 h at room temperature. At the same time, microtiter plates (Sigma) were incubated with 0.5 μg of desmosine-albumin conjugate (Elastin Product Company, Inc, Owensville, MO) in 0.05 M sodium carbonate buffer pH 9.6 at 4°C. After incubation, wells were washed five times with 0.05% Tween 20 in 0.15 M PBS, pH 7.2 and saturated with 0.05% Tween 20 in 0.15 M PBS, 1% BSA pH 7.2 for 1 h at room temperature. Eightfold aliquots of AbI-standard or AbI-sample solutions were then added to the wells for a 2 h incubation at room temperature. Wells were then in succession incubated with anti-rabbit IgG (1:2000) (Sigma) for 2 h at room temperature and with peroxidase-antiperoxidase complex (1:200) (Sigma) for 1 h at room temperature. 2,2'-Azino-bis (3-ethyl-benz-thiazoline-6-sulfonic acid) solution (Sigma) was then added to the wells. After incubation for 1 h at room temperature, absorbance was read at 405 nm. Data were expressed as μg/lung.

#### Morphology and Morphometry

The lungs from the different groups of mice were fixed by intra-tracheal inflation with buffered formalin (5%) at a constant pressure of 20 cm H_2_O at least for 24 hours. All lungs were then dehydrated, cleared in toluene, and embedded in paraffin. Sagittal sections (7 μm) of each pair of lungs were cut and stained with hematoxylin/eosin and Masson's trichrome stain. Morphometric assessment consisted of the determination by point counting, of the percent volume densities of fibrosis Vv(f) and of the emphysematous changes Vv(e) according to the stereological principle of Glagoleff and Weibel [22]: Vv = Pp, where Vv is the volume density and Pp the fraction of points superimposed a defined structural change. Point counting was performed at 100 × by determining 20 random fields per slide and using a multipurpose grid to count 45 points per field for a total of 900 points per slide. Fibrosis was defined as: "inflammatory and mesenchymal cell infiltration within the alveolar septa and alveolar spaces with deposition of extracellular matrix", and emphysematous changes were defined as: "abnormal enlargement of air spaces with loss of alveolar septa, and with or without thickening of the alveolar walls" [4].

The morphometric assessment of emphysema was also performed in all animals by determining the average inter-alveolar distance (mean linear intercept: Lm) [17]. For the determination of the Lm for each pair of lungs, 40 histological fields were evaluated both vertically and horizontally. Care was taken to avoid histological fields containing large bronchi, major vessels and areas of fibrosis.

#### Immunohistochemistry

For the immunohistochemical studies, tissue sections (8 μm) were stained for TGF-β, TGF-α and NE by an immunoperoxidase method. The sections were pre-treated with 3% hydrogen peroxide to inhibit the activity of the endogenous peroxidase. For antigen retrieval, the sections were heated in a microwave for 20 min in citrate buffer 0.01 M, pH 6.0, and allowed to cool slowly to room temperature. All the sections were incubated with 3% bovine serum albumin for 30 min at room temperature to block non-specific antibody binding. They were then incubated overnight at 4°C with the primary antibodies (Ab). The primary polyclonal Ab used were: rabbit Ab to mouse TGF-a diluted 1:50 (Santa Cruz Biotechnology, Inc., Santa Cruz, CA), rabbit Ab to mouse TGF-α diluted 1:20 (Insight Biotechnology LTD., Wembley, England). For elastase detection we used rabbit Ab to human neutrophil elastase (cross-reacting with mouse neutrophil elastase) diluted 1:500 (Calbiochem-Novabiochem, San Diego, CA). All the sections were rinsed and incubated with sheep anti-rabbit IgG for 30 min at room temperature. The staining was revealed by adding peroxidase-antiperoxidase complex, prepared from rabbit serum. Detection was accomplished by incubating in diamino-benzidine freshly dissolved in 0.03% H_2_O_2 _in 50 mM Tris-HCl pH 7.6. As negative controls for the immunostaining the primary antibodies were replaced by non-immunised rabbit serum. The sections were counterstained with hematoxylin.

#### Determination of elastase burden by immunoelectron microscopy

The immunogold method (post-embedding technique) was used to localize elastase in thin lung sections prepared for electron microscopy using anti-mouse neutrophil elastase (MNE) antibodies obtained as previously described [19]. Lung tissue blocks (5/animal) were taken from two animals from each group. The blocks, 1 to 2 mm in thickness, were fixed for 3 hours in 4% paraformaldehyde and 0.1% glutaraldehyde in 0.1 M phosphate buffer (pH 7.2), dehydrated in acetone, and embedded in epoxy resin (Araldite) without postfixation in OsO_4_. Ultrathin sections (600 Å thick) were picked up on nickel grids and pretreated with phosphate-buffered saline containing 1% ovalbumin for 5 minutes. The grids were then floated on a drop of diluted anti-MNE antibodies (1:2600) for 48 h at 4°C; the grids were thoroughly rinsed for 10 min with a mild spray of phosphate-buffered saline and then distilled water and transferred onto 15 μl drops of a protein A-gold particles (15 nm) (E-Y-LABS, San Mateo, CA) solution diluted 1:8 in phosphate-buffer saline. The sections were then washed, dried, stained with uranyl acetate-lead citrate, and examined in a Philips 300 electron microscopy. Ten to 12 micrographs (final magnification, × 12,000) were taken for each grid. To exclude false-positive labeling, a series of control studies (including also the use of nonimmune rabbit serum or of BSA instead of ovalbumin) were carried out as previously described in detail [19]. The density of gold particles per square micrometer of lung tissue was determined for each of the micrographs with a superimposed quadratic lattice grid. A total of 50 micrographs was thus analyzed for each animal, and the average of gold particle density on lung connective tissue of each group was calculated.

### Statistical Analysis

The significance of differences of the mean values was calculated using one-way ANOVA (F-test). A p value of less than 0.05 was considered significant.

## Results

### Bleomycin Study

#### Emphysema and Fibrosis after Bleomycin

**Figure 1:**
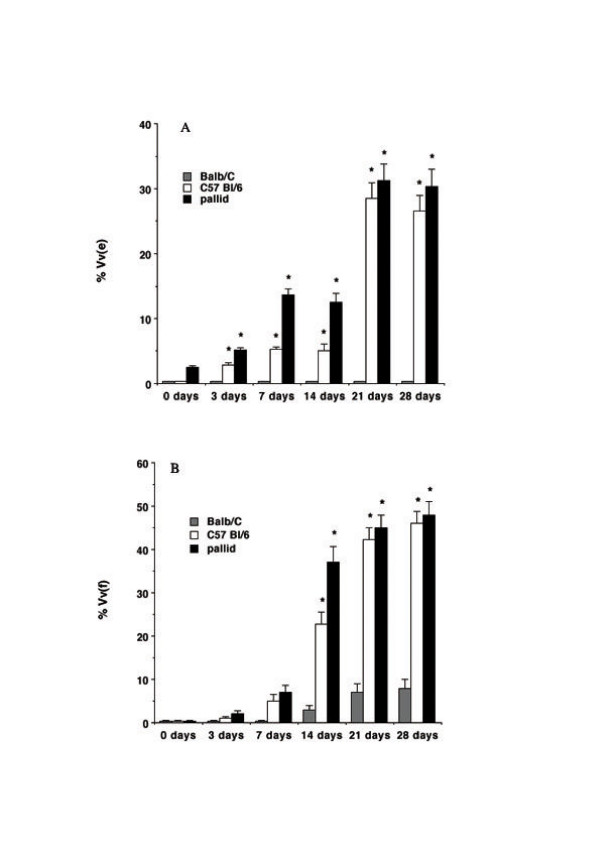
**Emphysema and fibrosis after bleomycin challenge. **The volume densities of emphysematous changes (Vv(e)) (A) and fibrosis (Vv(f)) (B) were quantitated by morphometry (point counting) on hematoxylin/eosin or Masson's trichrome stained lung sections, at various times after bleomycin. Data from 10 animals for each time point are given as mean ± SEM of per cent lung volume densities. *: p < 0.01 versus respective untreated controls (0 days).

The kinetics of the emphysematous and fibrotic changes obtained in the three strains of mice are reported in Fig 1 A and 1 B. In particular, we present the percent values of lung volume densities of fibrotic (Vv(f)) and emphysematous (Vv(e)) changes obtained at the various time points. In BLM-resistant *Balbc *mice with a normal antiprotease protection, negligible foci of cellular infiltration and fibrosis and no areas of air-space enlargements were detected during the period of the study. On the contrary, a progressive increase of areas of air space enlargement and fibrosis were seen in BLM-prone *C57 Bl/6 and pallid *mice with a mild and a marked deficiency of α1-PI, respectively.

**Figure 2:**
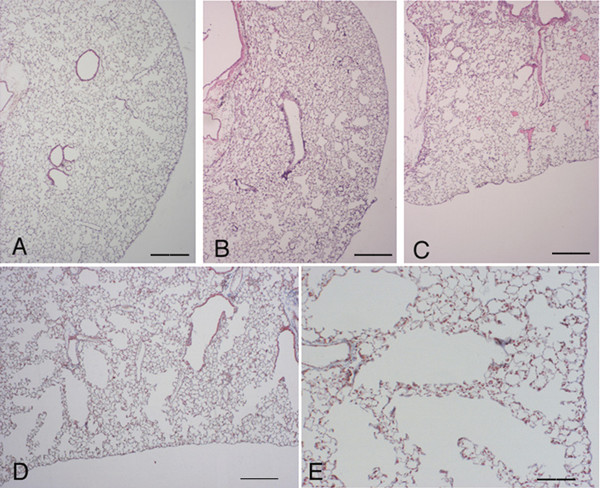
**Histological appearance of *C57 BI/6 *mouse lungs 3 and 7 days following bleomycin challenge. **(A) Histologic section from the lung of a *C57 Bl/6 *mouse treated with saline showing a normal parenchyma. Representative histologic sections of *C57 Bl/6 *mice at 3 (B) and 7 (C and D) days after bleomycin treatment showing appreciable morphologic emphysema but not fibrosis. Scattered inflammatory cells are present through lung parenchyma (E). (E) Shows a higher magnification of (D). (A-C): Hematoxylin-eosin stain, scale bar represents 400 μm. (D) and (E): Masson's trichrome stain, original magnification × 40 and × 100, respectively. Scale bars represent 400 μm and 100 μm, respectively.

**Figure 3:**
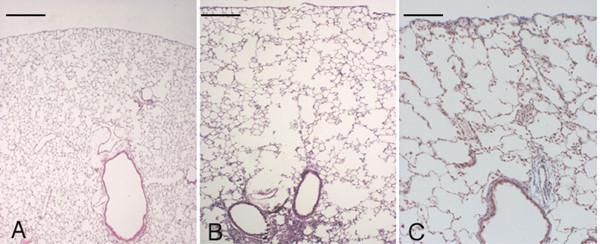
**Histological appearance of *pallid *mouse lungs 7 days following bleomycin challenge. **(A) Histologic section from the lung of a *pallid mouse *treated with saline showing a normal parenchyma. Lung sections *of pallid *mice at 7 days after bleomycin showing appreciable emphysema (B) and spotty areas of inflammatory cell infiltration without fibrosis (C). (A) and (B): Hematoxylin-eosin stain, scale bar represents 400 μm. (C): Masson's trichrome stain, scale bar represents 100 μm.

**Figure 4:**
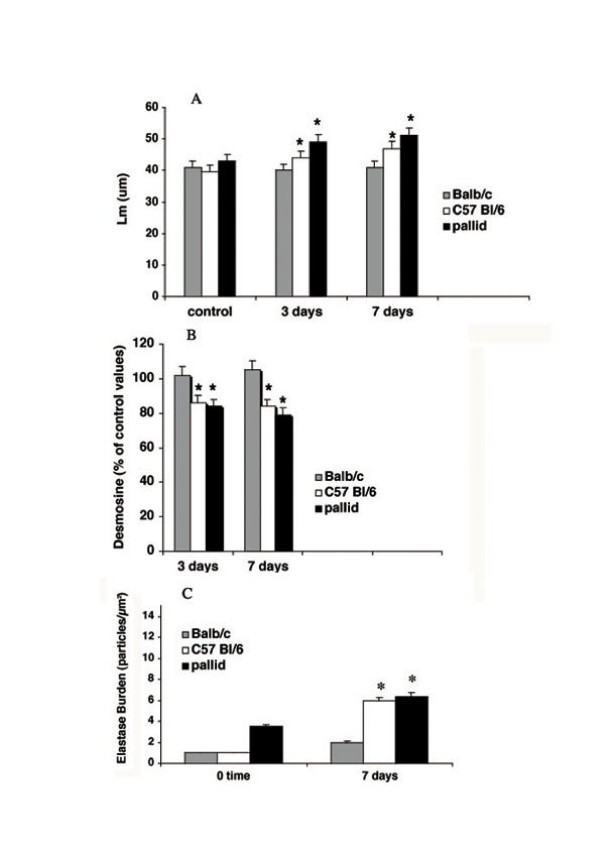
**Mean linear intercepts, lung desmosine and elastase burden in various strains of mice following bleomycin challenge. **(A) Mean linear intercepts (Lm) in *Balb/C, C57 Bl/6 *and *pallid *mice after bleomycin challenge. Data are from 10 animals for each time point and are given as mean ± SD. *: p < 0.01 versus respective saline-treated group. (B) Lung desmosine content in *Balb/C, C57 Bl/6 and pallid *mice after bleomycin treatment. Data from 10 animals for each time point are given as mean ± SD and represent per cent change over respective saline-treated controls *(Balb/C: *2.50 ± 0.28 μg/lung; *C57 BI/6: *2.48 ± 0.30 μg/lung; *pallid: *2.44 ± 0.32 μg/lung). *: p < 0.01 versus respective saline-treated group. (C) Lung elastase burden in *Balb/C, C57 Bl/6 and pallid *mice at 7 days after bleomycin treatment. Data are given as mean ± SD of the number of gold particles per μm^2^. *: p < 0.01 versus respective saline-treated group.

At 3 and 7 days after BLM, *C57 Bl/6 *and *pallid* mice showed appreciable morphologic emphysema with spotty areas of inflammatory cell infiltration in the absence of fibrotic changes (Figs 2 and 3). At these times the anatomical emphysema was associated with a significant increase of the Lm (Fig. 4 A) a significant decrease in lung desmosine content (Fig. 4 B) and a high neutrophil elastase burden (Fig. 4 C).

**Figure 5:**
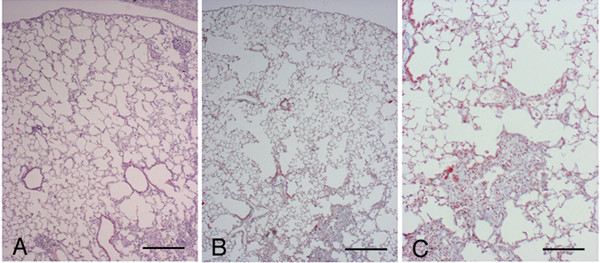
**Histological appearance of *pallid *and *C57 Bl/6 *mouse lungs 14 days following bleomycin challenge. **Representative lung histologic sections *of a pallid *(A) and a *C57BI/6 *(B) mouse at 14 days after bleomycin. Fibrotic and emphysematous areas are widely spread and intermixed. Emphysema is often located quite distant from the fibrotic reaction (A-B).(C) shows a higher magnification of (B). (A): Hematoxylin-eosin stain, scale bar represents 400 μm. (B) and (C): Masson's trichrome stain, scale bars represent 400 μm and 100 μm, respectively.

At 14 days after BLM, air spaces enlargements affected 6,68 ± 3.11 % and 12.71 ± 4.17 % (p < 0.01) of lung in *C57Bl/6 *and *pallid *mice, respectively (Fig. 1 A). At this time, the lungs of *C57 Bl/6 *and *pallid *mice showed also large areas of fibrosis which involved 24.11 ± 8.81 and 36.84 ± 11.47 % of the lungs, respectively (Fig. 1 B). Both lesions were widely spread and intermixed (Fig. 5 A). Nevertheless, areas of emphysema could also be detected in lung lobes without any fibrotic reaction. Additionally, in several areas the emphysematous changes were distant from the fibrotic foci (Fig. 5 B and 5 C).

**Figure 6:**
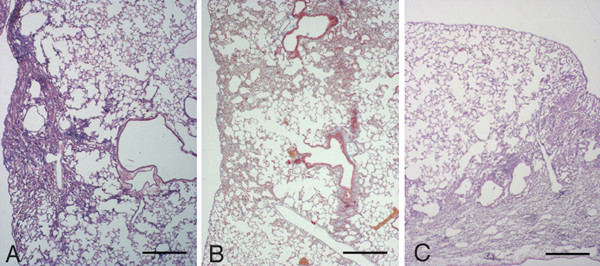
**Histological appearance of *C57 BI/6 *and *pallid *mouse lungs 21 days following bleomycin challenge. **Histologic sections from lungs of a *C57BI/6 *(A and C) and *pallid *(B) mouse at 21 days after bleomycin. The emphysematous lesions within or adjacent to the fibrotic areas appear to be mainly of the paracicatricial type (A and B). Nevertheless, several areas of emphysema can be detected quite distant from the fibrotic zones (C). (A) and (C): Hematoxylin-eosin stain, scale bar represents 400 μm. (B): Masson's trichrome stain, scale bar represents 400 μm.

At later times (21 days onward), the volume density of emphysematous and fibrotic changes markedly increased (Fig. 1 A and 1 B). From a morphological point of view, the emphysematous lesions appeared to be mainly of paracicatricial type (Fig. 6 A and 6 B). Nevertheless, large areas of emphysema could also be found in lung lobes without obvious fibrosis or situated adjacent to the fibrotic lesions (Fig. 6 C).

No significant changes in terms of emphysema or fibrosis were seen after BLM treatment in *Balb/c *mice with normal levels of serum αl-PI, by morphological, morphometrical and biochemical analysis.

#### Immunohistochemistry

**Figure 7:**
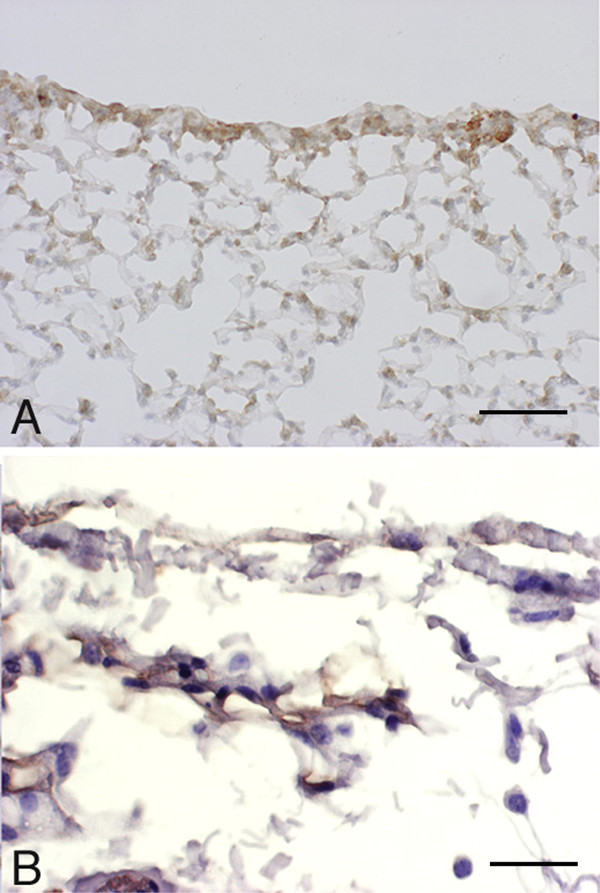
**Immunohistochemical reaction for TGF-β and TGF-α 7 days following bleomycin challenge. **Lung parenchyma of a *C57BI/6 *mouse at 7 days after bleomycin treatment. (A) Immunohistochemical reaction for TGF-β. Counterstained with hematoxylin, scale bar represents 40 μm. (B) Immunohistochemical reaction for TGF-α. Counterstained with hematoxylin, scale bar represents 25 μm.

Lungs of mice from the three strains were also analysed for cytokine expression by immuno-histochemistry. A significant change of some cytokines related to the NE activity was observed in mice with a mild *(C57 Bl/6) *and a marked deficiency *(pallid) *of αl-PI, after BLM administration. In particular, TGF-β was detected in subpleural foci of cellular proliferation at 7 days (Fig. 7 A) when an increase of the elastase burden could also be demonstrated. Also at 7 days, an evident staining for TGF-α was observed in subpleural and peribronchiolar areas (Fig. 7 B).

#### Effects of a serine proteinase inhibitor treatment on BLM-induced lesions

**Figure 8:**
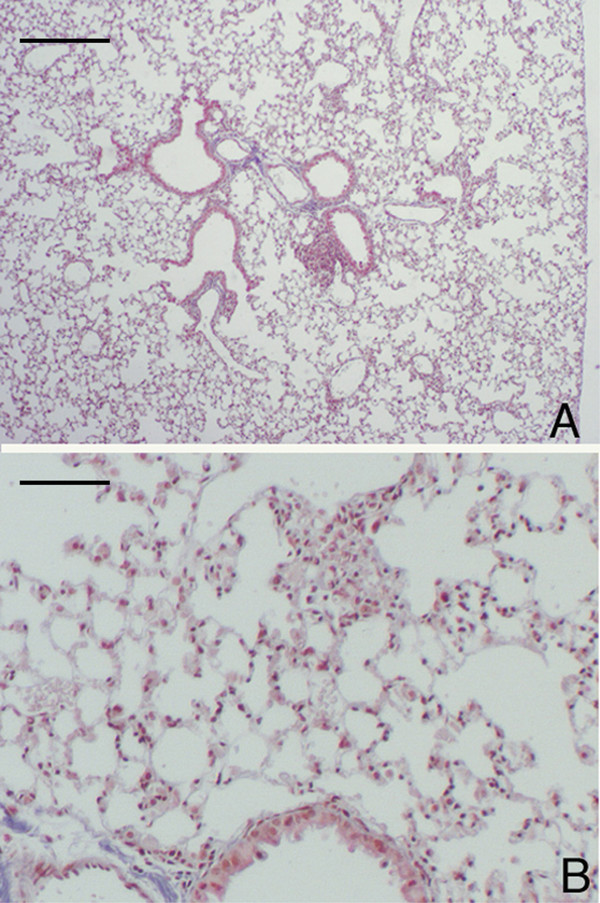
**Histological appearance of C57 *BI/6 *lung receiving 4-(2-aminoethyl)-benzenesulfonyl fluoride hydrochloride 21 days after bleomycin challenge. **Representative histological section of a *C57BI/6 *mouse, receiving 4-(2-aminoethyl)-benzenesulfonyl fluoride hydrochloride and bleomycin, at 14 days after the treatment. No appreciable areas of emphysema are detectable in the lung parenchyma (A). Few trivial foci of fibrosis can be appreciated in some areas (B). (B) shows a higher magnification of (A). (A) and (B): Masson's trichrome stain, scale bars represent 400 μm and 50 μm, respectively.

The treatment of animals with 4-(2-aminoethyl)-benzenesulfonyl fluoride hydrochloride, a serine-proteinase inhibitor, significantly prevented the BLM-induced lesions in *C57 Bl/6 *mice. In particular, treated animals showed 14 days after BLM treatment no areas of emphysema and only trivial foci of fibrotic reaction (Fig. 8 A, and 8 B). The Lm values (40.75 ± 0.91 μm) and the lung (Vv(e)) (0.93 ± 0.89 %) in these mice were not significantly different from those observed in control mice (Lm: 39.71 ± 0.80 μm; Vv(e): 0.26 ± 0.74 %). In addition the lung (Vv(f)) (14.13 ± 6.21 %) in mice receiving BLM plus proteinase inhibitor was significantly lower (p < 0.01) than that observed in mice receiving only BLM (24.11 ± 9.41 %).

No immunological reaction for TGF-α and a faint positive staining TGF-β was found 7 days after BLM administration in animals treated with 4-(2-aminoethyl)-benzenesulfonyl fluoride hydrochloride (data not shown).

### Cigarette Smoke Study

**Figure 9:**
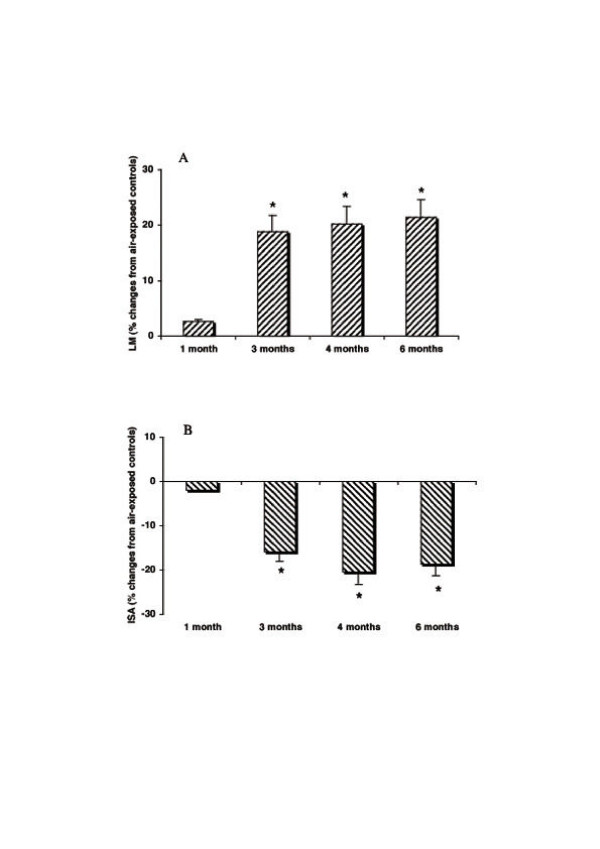
**Mean linear intercepts and lung internal surface areas of *DBA/2 *mice at various time-points during chronic cigarette smoke exposure. **Mean linear intercept (LM) (A) and internal surface area /ISA) (B) of the lungs of *DBA/2 *mice at various time-intervals during chronic exposure to cigarette smoke. Data from 8 animals for each time point are given as mean ± SD. * p < 0.05 versus air-exposed controls.

**Figure 10:**
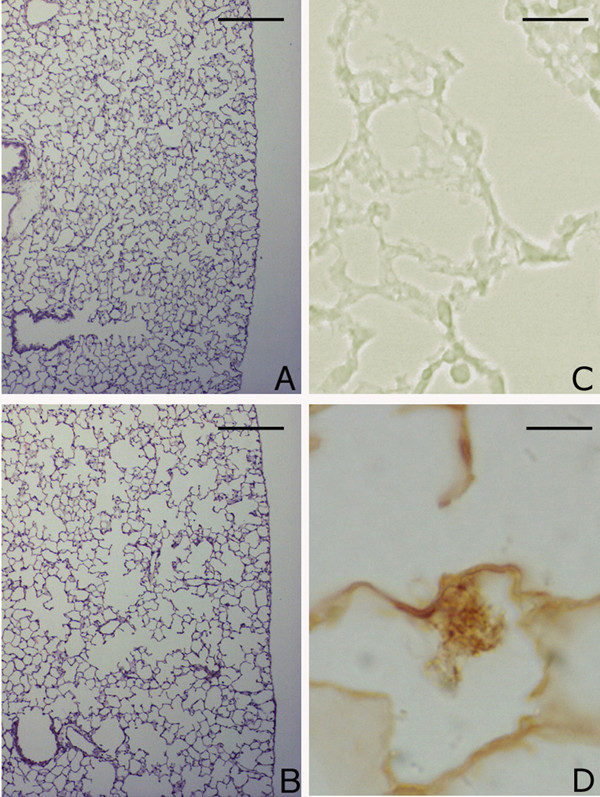
**Lung histology and immunohistochemical reaction for neutrophil elastase in *DBA/2 *mice 3 months after chronic cigarette smoke exposure. **Lung parenchyma from an air-exposed (A) and a smoke-exposed *DBA/2 *(B) mouse, at 3 months. Hematoxylin-eosin stain, scale bar represents 400 μm. (C) and (D): Immunohistochemical reaction for neutrophil elastase on alveolar septa of DBA/2 mouse at 3 months after cigarette smoke exposure in absence (C) or in presence of the primary antibody (D). Scale bar represents 25 μm.

**Figure 11:**
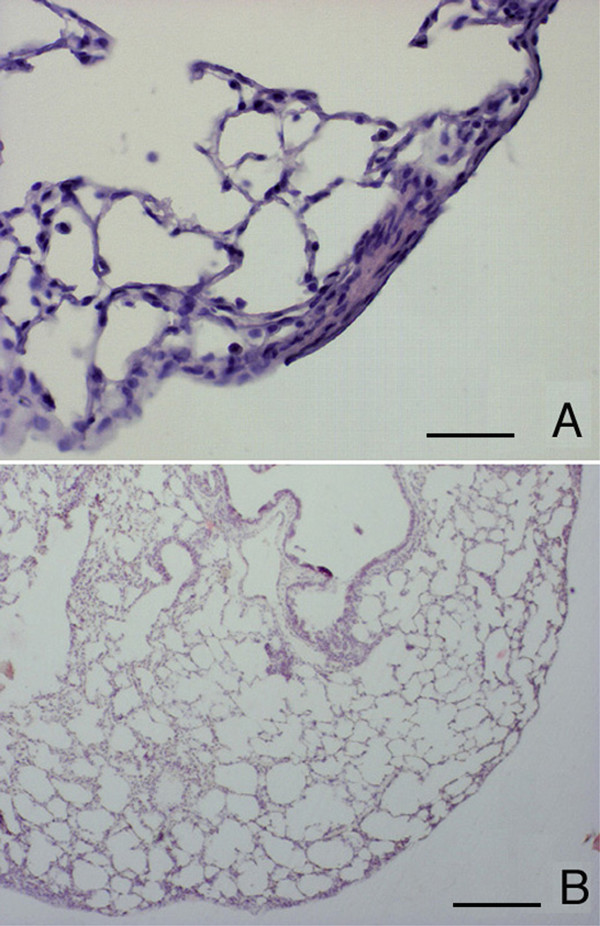
**Histological appearance of *DBA/2 *lungs 4 and 6 months after chronic cigarette smoke exposure. **Lung parenchyma from smoke-exposed *DBA/2 *mice, at 4 (A) and 6 months (B). The first foci of subpleural fibrosis are seen from 4 months of smoke exposure (A). After 6 months of cigarette smoke exposure, disseminated foci of severe emphysema and evident areas of subpleural fibrosis are present (B). (A) and (B): Hematoxylin-eosin stain, Scale bars represent 25 μm and 400 μm. respectively.

The results of the morphometric assessment at various time points are shown in Fig. 9. Already at 3 months of smoke exposure the *DBA/2 *mice showed overt emphysema (Fig. 10 B) characterized by significant changes both of the Lm (+ 19 %, p < 0.01) and of the internal surface area (ISA) (-16 %, p < 0.01) (Fig.9). At this time, immunohistochemical examination revealed a positive reaction for mouse NE on the alveolar septa (Fig. 10 C). Of interest, the first foci of fibrosis were seen after 4 months of smoke exposure (Fig. 11 A) and their severity progressively increased with time reaching at 6 months a (Vv(f)) value of 5.53 ± 2.11 %. At 6 months after smoke exposure, the fibrotic lesions consisted mainly of subpleural foci. In some areas, the fibrotic reaction was seen in the lung parenchyma associated or not with foci of emphysema (Fig. 11 B).

**Figure 12:**
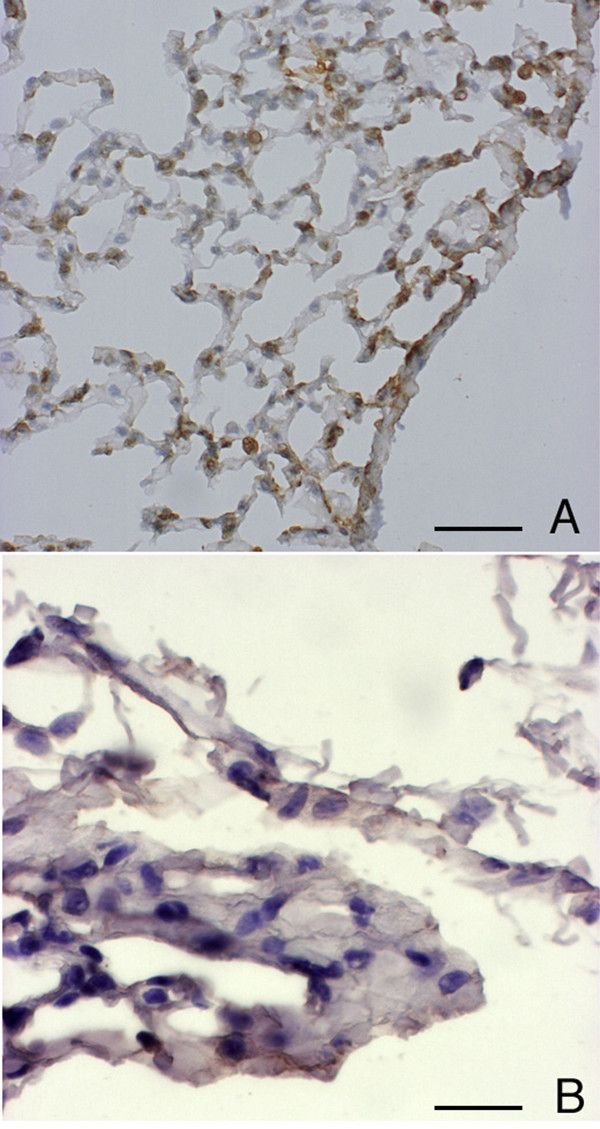
**Immunohistochemical reaction for TGF-β and TGF-α 3 months after chronic cigarette smoke exposure. **Lung parenchyma of a smoke-exposed *DBA/2 *mouse, at 3 months. (A) Immunohistochemical reaction for TGF-β. Counterstained with hematoxylin, scale bar represents 40 μm. (B) Immunohistochemical reaction for TGF-α. Counterstained with hematoxylin, Scale bar represents 15 μm.

A positive immunohistochemical reaction for TGF-β and TGF-α could be demonstrated starting from 3 months of smoke exposure onwards (Figs. 12 A and 12 B). In general, these cytokines were detected in foci of cellular proliferation and subsequently (from 4 months onward) in subpleural and parenchymal areas of fibrosis.

## Discussion

Although lung emphysema and fibrosis may result from two distinct and apparent opposite processes, they may coexist either in different areas, or in a same area of the lung of humans and animals. The development and the degree of these morphological responses that generally follow, or exacerbate, an acute or chronic inflammation can be influenced by many individual factors, such as cytokine production, variation in collagen synthesis and deposition, antiprotease screen and antioxidant status [23-30].

The findings reported in this paper strongly suggest that NE may represent a common factor affecting the development of both emphysema and fibrosis.

In particular, we demonstrate that in mice BLM-treated which are genetically deficient in αl-PI (*i*) emphysema and fibrosis may coexist either in different areas, or in a same lung area; (*ii*) the development of emphysema precedes that of fibrosis; (*iii*) the development of emphysematous lesions, shortly after BLM administration, is preceded by an alveolar elastolytic burden and is matched by a marked decrease in lung desmosine content; and finally, (*iiii*) an evident staining for TGF-β and TGF-α is observed when an increased neutrophil elastase burden can also be demonstrated. Similarly, we found in lungs of mice after cigarette-smoke exposure that (*i*) emphysema and fibrosis may be present in the same lung; (*ii*) the development of the emphysematous lesions occur at earlier time points than that of the fibrotic foci, and *(iii) *a positive immunohistochemical reaction for neutrophil elastase is associated with a positive reaction for TGF-β and TGF-α two major fibrogenic cytokines (i.e. [31,32] in foci of cellular proliferation, and in areas of fibrosis.

Taken all together these results indicate that the air-space enlargements observed in mice with a genetic deficiency of serum α_l_-PI, early after BLM, represent areas of "true" emphysema caused by a proteolytic attack and characterised by lung desmosine loss. The strong immunoelectron microscopical reaction for NE found on alveolar septa of α_l_-PI deficient mice early after BLM and in DBA/2 mice after cigarette smoke suggests that NE may represent a common factor affecting the development of both emphysema and fibrosis.

This hypothesis is further supported by the data obtained in BLM-treated C57 B1/6J mice in which both emphysema and fibrosis were significantly attenuated by the use of a serine proteinase inhibitor active against NE. Of interest, in these animals no immunological reaction for TGF-α and only a faint positive staining for TGF-β could be demonstrated. Although there is no ideal animal model, including the BLM one, that mimics human idiopathic pulmonary fibrosis, the data reported here support a role for proteases, and in particular for NE, in both these two pathologies.

It is well know that proteases released by inflammatory cells recruited at the site of inflammation may be involved in the intracellular as well as extracellular route of catabolism of interstitial proteins. These proteases may play an important role in tissue injury and repair by degrading the components of the extracellular matrix [33]. During the reparative responses they can remove scar tissue influencing in this way the morphological end-point.

It is generally accepted that NE plays a role in the development of emphysematous lesions. It acts on a large variety of substrates, in particular elastin, collagens, fibronectin, laminin and proteoglycans [23]. Recent studies suggest that this enzyme may modulate the fibrotic response also by interacting with the cytokine network. NE can activate or inactivate by proteolytic cleavage several cytokines, receptors and polypeptide growth factors implicated in inflammation and reparative phases of the fibrotic response [34-37].

In this regard, NE constitutes an important factor for the generation of soluble TGFα [32], a potent mitogenic cytokine for mesenchymal cells. In fact, TGFα is activated by the cleavage of the membrane precursor pro-TGFα by elastase [38,39].

Additionally, NE modulates TGF-β bioactivity [35] either directly by releasing TGF-β1 from the extracellular matrix [40] or indirectly *via *MMP-12 [41].

Of interest, we found that TGFα and TGFβ immunoreactivity was significantly high in mice that develop foci of subpleural fibrosis after cigarette-smoke or BLM treatment when a positive reaction for mouse NE on the alveolar septa was found. Although the association between the development of foci of subpleural fibrosis and a positive reaction for mouse NE on the alveolar septa does not prove a causal relationship, the data presented here strongly support the hypothesis that NE may represent a common pathogenic link between emphysema and fibrosis.

## Conclusion

In conclusion the data reported in this paper strongly suggest a significant role for the NE not only in the development of pulmonary emphysema, but also in the modulation of fibrotic lesions. The results may also offer an explanation for the antifibrotic activity of some protease inhibitors (i.e. α_l_-PI, SLPI and the synthetic inhibitor ONO-5046) that are all active against NE.

## Competing interests

The author(s) declare that they have no competing interests.

## Authors' contributions

ML played a major role in the design of the study, acquisition, analysis and interpretation of data and drafting the manuscript. BB and BL performed the histological and morphometrical analyses, and contributed to the interpretation of the results. EC and SF performed histochemical and biochemical analyses, carried out animal studies and participated in the interpretation of data. PAM performed some of the morphometrical analysis and contributed to the interpretation of data. GL conceived and coordinated the study, participated in the design of the study, analysis and interpretation of data and drafting the manuscript. All authors read and approved the final manuscript.
